# A dichotomy color quantization algorithm for the HSI color space

**DOI:** 10.1038/s41598-023-34977-0

**Published:** 2023-05-19

**Authors:** Xia Yu, Huaiyu Zhuang, Yani Cui, Jiaxian Deng, Jia Ren, Haixia Long

**Affiliations:** 1 College of Information and Communication, Hainan University, Haikou, 570100 Hainan China; 2 College of Information Science and Technology, Hainan Normal University, Haikou, 570100 Hainan China; 3 China Mobile Financial Technology Co., Ltd., Beijing, 100011 China

**Keywords:** Mathematics and computing, Information technology, Engineering, Electrical and electronic engineering

## Abstract

Color quantization is used to obtain an image with the same number of pixels as the original but represented using fewer colors. Most existing color quantization algorithms are based on the Red Green Blue (RGB) color space, and there are few color quantization algorithms for the Hue Saturation Intensity (HSI) color space with a simple uniform quantization algorithm. In this paper, we propose a dichotomy color quantization algorithm for the HSI color space. The proposed color quantization algorithm can display images with a smaller number of colors than other quantization methods of RGB color space. The proposed algorithm has three main steps as follows: first, a single-valued monotonic function of the Hue (H) component in the from RGB color space to HSI color space (RGB-HSI) color space conversion is constructed, which can avoid the partition calculation of the H component in the RGB-HSI color space; second, an iterative quantization algorithm based on the single-valued monotonic function is proposed; and third, a dichotomy quantization algorithm is proposed to improve the iterative quantization algorithm. Both visual and numerical evaluations reveal that the proposed method presents promising quantization results.

## Introduction

Color quantization methods define a quantized palette with fewer colors than the original palette. After this, a quantized image is obtained by representing each pixel of the original image using a color of the quantized palette in such a way that the new image is as similar as possible to the original image. Reducing the number of different colors of an image allows it to be displayed on low-end devices. In addition, the size of the image is reduced, which improves the image transmission speed and optimizes the storage space^1^.

Most color quantization algorithms are based on the Red Green Blue (RGB) color space^2–6^, and the most popular color quantization algorithm is the K-means clustering scheme, which is widely applied to color quantization^7^.

Omran^8^ proposed a color quantization method combining a particle swarm optimization algorithm with K-means (PSO + KM), Ozturk^9^ combined an artificial bee colony (ABC) algorithm, and Pérez-Delgado proposed various color quantization algorithms^10–12^. In Ref.^13^, a particle swarm optimization algorithm and ant tree were combined to solve the problem of color quantization. Reference^14^ proposed a fast color image quantization algorithm including initial palette generation and fast K-means clustering. The iterative ant-tree for color quantization (ITATCQ) algorithm was a recently proposed method of this type^15^ based on the ant-tree for color quantization (ATCQ) algorithm^16^. Although most color quantization algorithms are based on the RGB color space, the mean square error (MSE) value of reconstructed images using these algorithms is not low.

Since the components of the RGB color space are not independent of each other, the distortion of a single component will cause distortion of the other components during image processing^17^. In addition, conversion to the Hue Saturation Intensity (HSI) space is beneficial for obtaining better quality decompressed images, and the PSNR is higher in the HSI color space than in the RGB color space^18^. Many scholars transfer images from the RGB color space to the HSI color space^19–24^, the calculation of color quantization algorithms in HSI space is more complex compared to other color spaces, requiring the conversion and processing of color components, so there are few color quantization algorithms for the HSI space, the quantization methods are simple, the quantization of H, S, I components are uniformly selected^25^.

Therefore, we propose a new dichotomy color quantization algorithm for the HSI color space, which displays images with a smaller number of colors. First, the Hue (H) component’s piecewise function is mapped to construct a single-valued monotonic function, and the H component can be expressed as a single-valued monotonic function. Second, we propose an iterative quantization algorithm based on this single-valued monotonic function. Third, we propose a dichotomy quantization algorithm to improve the iterative quantization algorithm.

The main contributions of this paper can be summarized as follows:

(i) This proposed quantization algorithm provides a new idea for color quantization methods, which can be used by researchers in HSI color space. (ii) We map the H component of the HSI color space to a single-valued monotonic function, which can be used for quantization. (iii) We propose iterative quantization algorithm and dichotomy quantization algorithm of the HSI color space.

## Methods

In this section, part A, C, and D are the solutions described corresponds to our contribution, part B is taken from other existing methods. In part A, we map the H component of the HSI color space to a single-valued monotonic function, and then according to the optimized quantization algorithm of part B, we propose an iterative quantization algorithm of part D, and the last we propose a dichotomy quantization algorithm to improve the iterative quantization algorithm. The solution of part D is better than the solution of part C, so we choose dichotomy quantization algorithm of part D to compare with other quantization algorithms.

### A. The single-valued monotonic function transformation of the H component

**Figure 1:**
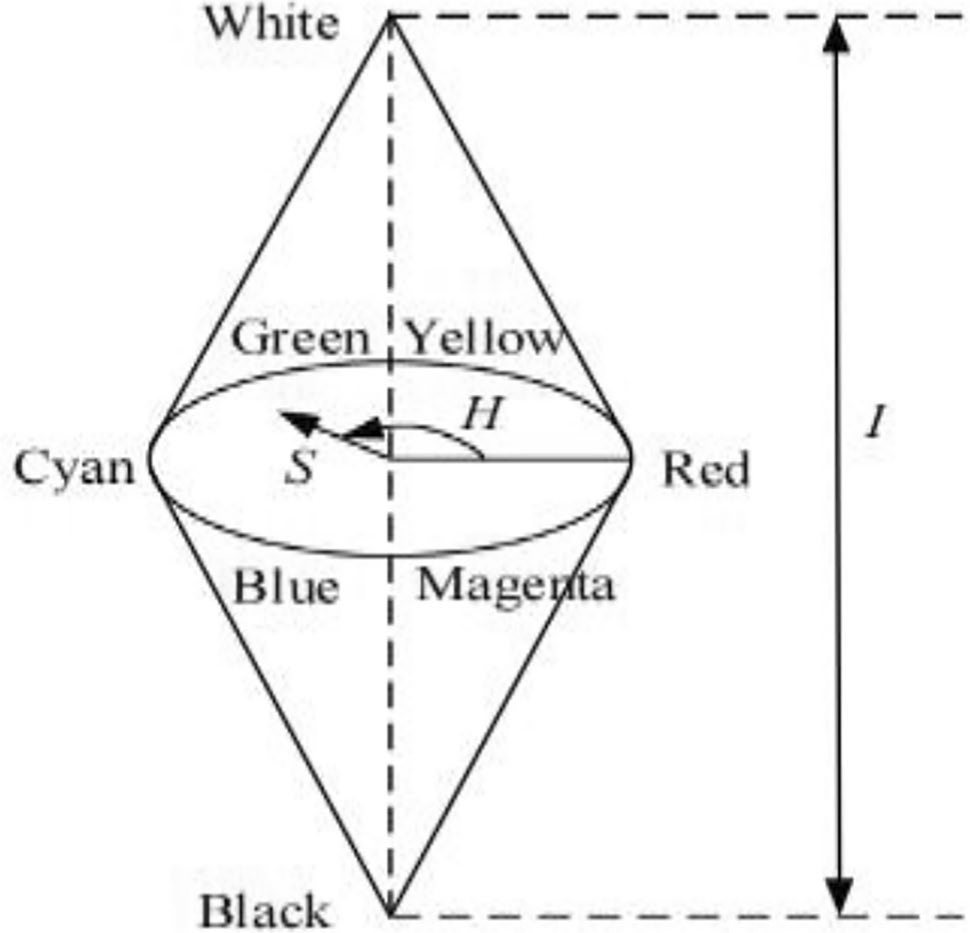
Model of the HSI color space.

**Figure 2:**
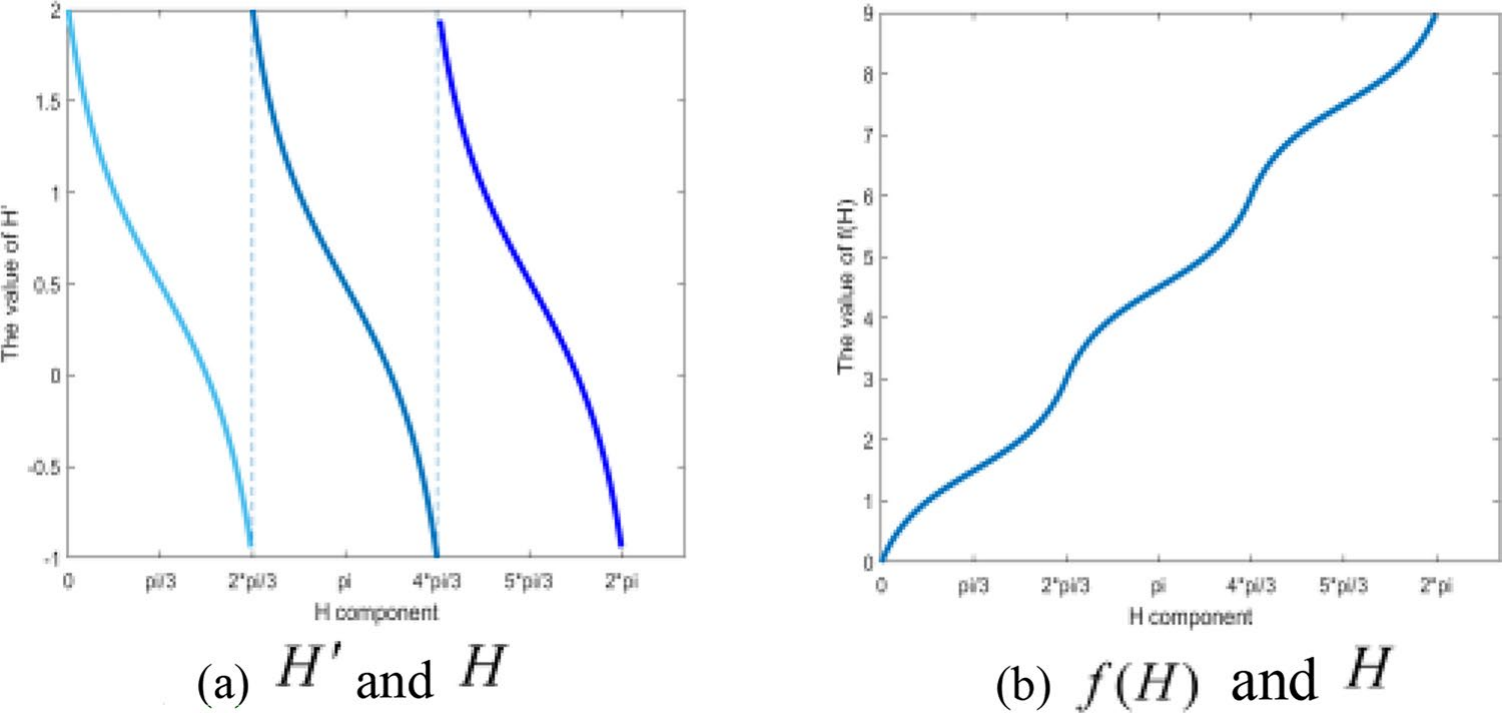
The relationship of $H^{\prime}$ and $H$*,* $f(H)$ and $H$.

- The model of the HSI color space is shown in Fig. 1. The hue component H has three different sectors $[0,{{2\pi } \mathord{\left/ {\vphantom {{2\pi } 3}} \right. \kern-0pt} 3})$, $[{{2\pi } \mathord{\left/ {\vphantom {{2\pi } 3}} \right. \kern-0pt} 3},{{4\pi } \mathord{\left/ {\vphantom {{4\pi } 3}} \right. \kern-0pt} 3})$ and $[{{4\pi } \mathord{\left/ {\vphantom {{4\pi } 3}} \right. \kern-0pt} 3},2\pi )$.The article^26^ proposed a simple space conversion (S-SC) algorithm, and a new hue component $H^{\prime}$ is used for replacing the H component, thus the calculation of H component turns complex operations such as cosine into simple addition and subtraction operations, the $H^{\prime}$ can be calculated by ${{\cos \left( H \right)} \mathord{\left/ {\vphantom {{\cos \left( H \right)} {\cos \left( {{\pi \mathord{\left/ {\vphantom {\pi 3}} \right. \kern-0pt} 3} - H} \right)}}} \right. \kern-0pt} {\cos \left( {{\pi \mathord{\left/ {\vphantom {\pi 3}} \right. \kern-0pt} 3} - H} \right)}}$ and the simple operation of $H^{\prime}$ is shown as Eq. (1). The relationship between the hue components $H^{\prime}$ and $H$ is shown in Fig. 2(a). It can be seen from the Fig. 2(a) that the value of $H^{\prime}$ is not discontinuous on the interval $[0,2\pi )$, and the quantization operation cannot be performed for hue component $H^{\prime}$_._
  1 $$H^{\prime} = \left\{ {\begin{array}{*{20}l} {\frac{{2{\text{R}} - {\text{G}} - {\text{B}}}}{{{\text{R}} + {\text{G}} - 2{\text{B}}}}, H \in \left[ {0,2{\uppi }/3} \right]} \\ {\frac{{2{\text{G}} - {\text{B}} - {\text{R}}}}{{{\text{G}} + {\text{B}} - 2{\text{R}}}},H \in \left[ {2{\uppi }/3,{ }4{\uppi }/3} \right]} \\ {\frac{{2{\text{B}} - {\text{R}} - {\text{G}}}}{{{\text{R}} + {\text{B}} - 2{\text{G}}}},H \in \left[ {4{\uppi }/3,{ }2{\uppi }} \right]} \\ \end{array} } \right.$$

In the RGB color space to HSI color space conversion process, $${{\cos \left( H \right)} \mathord{\left/ {\vphantom {{\cos \left( H \right)} {\cos \left( {{\pi \mathord{\left/ {\vphantom {\pi 3}} \right. \kern-0pt} 3} - H} \right)}}} \right. \kern-0pt} {\cos \left( {{\pi \mathord{\left/ {\vphantom {\pi 3}} \right. \kern-0pt} 3} - H} \right)}}$$ is used instead of the H component in the S-SC algorithm^26^, and the value range of $$H^{\prime}$$ is $$[ - 1,2)$$ in sectors $$[0,{{2\pi } \mathord{\left/ {\vphantom {{2\pi } 3}} \right. \kern-0pt} 3})$$, $$[{{2\pi } \mathord{\left/ {\vphantom {{2\pi } 3}} \right. \kern-0pt} 3},{{4\pi } \mathord{\left/ {\vphantom {{4\pi } 3}} \right. \kern-0pt} 3})$$ and $$[{{4\pi } \mathord{\left/ {\vphantom {{4\pi } 3}} \right. \kern-0pt} 3},2\pi )$$. The relationship of $$H^{\prime}$$ and H component can be expressed in Fig. 2(a), the abscissa represents the H component, and the ordinate represents $$H^{\prime}$$. There are three segmented curves in Fig. 2(a), after inverting the three segmented curves and performing the shift operation, we can obtain the curves in Fig. 2(b), i.e., the relationship between $$f(H)$$ and *H* is shown in Fig. 2(b), the abscissa still represents the H component, and the ordinate is represented by the function $$f(H)$$.Therefore, function $$f(H)$$ can be constructed, and the value ranges of the constructed function for the three sectors are $$[0,3)$$, $$[3,6)$$ and $$[6,9)$$, respectively. Therefore, the value range of function $$f(H)$$ in the interval $$[0,2\pi )$$ is $$[0,9)$$. Function $$f(H)$$ is shown as formula (2), and the relationship between $$f(H)$$ and *H* is shown in Fig. 2(b).

2 $$f(H) = \left\{ {\begin{array}{*{20}l} {2 - \frac{\cos H}{{\cos ({\pi \mathord{\left/ {\vphantom {\pi 3}} \right. \kern-0pt} 3} - H)}},} \hfill & {H \in [0,{{2\pi } \mathord{\left/ {\vphantom {{2\pi } 3}} \right. \kern-0pt} 3})} \hfill \\ {5 - \frac{{\cos (H - {{2\pi } \mathord{\left/ {\vphantom {{2\pi } 3}} \right. \kern-0pt} 3})}}{\cos (\pi - H)},} \hfill & {H \in [{{2\pi } \mathord{\left/ {\vphantom {{2\pi } 3}} \right. \kern-0pt} 3},{{4\pi } \mathord{\left/ {\vphantom {{4\pi } 3}} \right. \kern-0pt} 3})} \hfill \\ {8 - \frac{{\cos (H - {{4\pi } \mathord{\left/ {\vphantom {{4\pi } 3}} \right. \kern-0pt} 3})}}{{\cos ({{5\pi } \mathord{\left/ {\vphantom {{5\pi } 3}} \right. \kern-0pt} 3} - H)}},} \hfill & {H \in [{{4\pi } \mathord{\left/ {\vphantom {{4\pi } 3}} \right. \kern-0pt} 3},2\pi )} \hfill \\ \end{array} } \right.$$

We have proved that function $f(H)$ is a monotonically increasing and continuous function in the interval $[0,2\pi )$, and the proof is shown in Online Supplementary Information.

### B. The optimized quantization algorithm

Assuming that the value range of a variable $x$ is $[a,b]$, $f(x)$ is the function of variable $x$, and $p(x)$ is the probability density function of $f(x)$. The function $f(x)$ is quantized into $N$ levels on the interval $[a,b]$. The minimum mean square error is used for the criterion, and the optimized quantization can be expressed as follows:

3 $$S^{2} = \sum\limits_{n = 0}^{N - 1} {\int_{{x_{n} }}^{{x_{n + 1} }} {[f(x) - y_{n} ]^{2} } } p(x)dx$$

where $x_{0} = a$, $x_{N} = b$ and $y_{n}$ is the quantized value of function $f(x)$ in the quantization interval $[x_{n} ,x_{n + 1} ]$. In particular, when variable $x$ obeys an equal probability distribution, Eq. (3) can be reduced to Eq. (4):

4 $$S^{2} = \sum\limits_{n = 0}^{N - 1} {\int_{{x_{n} }}^{{x_{n + 1} }} {[f(x) - y_{n} ]^{2} } } dx$$

Clearly, there are *N *− 1 unknown variables in sequence $x_{1} ,x_{2} \cdots ,x_{{N{ - }1}}$, there are *N* unknown variables about $y_{n}$,$n = 1,2, \cdots ,n$, and there are $2N - 1$ unknown variables in total. When $f(x)$ is a continuous differentiable function, the quantization interval $x_{n}$ and the corresponding quantized value $y_{n}$ can be obtained by calculating the derivative. The derivative of function $S^{2}$ to $x_{n}$ is taken, and by setting $\frac{{dS^{2} }}{{dx_{n} }} = 0$, we can obtain:

5 $$y_{n + 1} + y_{n} = 2f(x_{n} )$$

In Eq. (5), the value range of $n$ is $n = 0,1,2 \cdots ,N - 2$, and there are $N - 1$ equations in Eq. (4). The derivative of function $S^{2}$ to $y_{n}$ is taken, and by setting $\frac{{dS^{2} }}{{dy_{n} }} = 0$, we can obtain:

6 $$y_{n} [x_{n + 1} - x_{n} ] = \int_{{x_{n} }}^{{x_{n + 1} }} {f(x)} dx$$

Formula (6) can be transformed into:

7 $$y_{n} = \frac{{\int_{{x_{n} }}^{{x_{n + 1} }} {f(x)dx} }}{{x_{n + 1} - x_{n} }}$$

It can be seen from Eq. (7) that the quantized value $y_{n}$ is the median value of function $f(x)$ in the interval $[x_{n} ,x_{n + 1} ]$, and the value range of $n$ is $[0,N - 1]$ of $y_{n}$, so there are N equations in Eq. (7). In summary, there are $2N - 1$ unknown variables in Eqs. (5) and (7), and the number of equations is also $2N - 1$, so the solution to the equation is unique. Substituting Eq. (7) into Eq. (5), we can obtain:

8 $$2f(x_{n} ) = \frac{{\int_{{x_{n - 1} }}^{{x_{n} }} {f(x)dx} }}{{x_{n} - x_{n - 1} }} + \frac{{\int_{{x_{n} }}^{{x_{n + 1} }} {f(x)dx} }}{{x_{n + 1} - x_{n} }}$$

where $$n = 0,1,2 \cdots ,N - 1$$.

9 $$\begin{gathered} f(x_{1} ) = \frac{1}{2}\left( {\frac{{\int_{{x_{0} }}^{{x_{1} }} {f(x)dx} }}{{x_{1} - x_{0} }} + \frac{{\int_{{x_{1} }}^{{x_{2} }} {f(x)dx} }}{{x_{2} - x_{1} }}} \right) \hfill \\ f(x_{2} ) = \frac{1}{2}\left( {\frac{{\int_{{x_{2} }}^{{x_{3} }} {f(x)dx} }}{{x_{2} - x_{1} }} + \frac{{\int_{{x_{2} }}^{{x_{3} }} {f(x)dx} }}{{x_{3} - x_{2} }}} \right) \hfill \\ \ldots \hfill \\ f(x_{n} ) = \frac{1}{2}\left( {\frac{{\int_{{x_{n - 1} }}^{{x_{n} }} {f(x)dx} }}{{x_{n} - x_{n - 1} }} + \frac{{\int_{{x_{n} }}^{{x_{n + 1} }} {f(x)dx} }}{{x_{n + 1} - x_{n} }}} \right) \hfill \\ \end{gathered}$$

When $x_{n - 1}$, $x_{n}$ are known, Eq. (9) can be transformed into:

10 $$K = 2f(x_{n} ) - \frac{{\int_{{x_{n - 1} }}^{{x_{n} }} {f(x)dx} }}{{x_{n} - x_{n - 1} }}$$

where *K* is a variable. To calculate $x_{n + 1}$, we can establish the equation: $g(x) = \frac{{\int_{{x_{n} }}^{x} {f(t)dt} }}{{x - x_{n} }}$. Here, *t* is only a variable, and the value range of *t* is between $x$ and $x_{n}$. Equation (9) can be expressed as:

11 $$g(x_{n + 1} ) = 2f(x_{n} ) - \frac{{\int_{{x_{n - 1} }}^{{x_{n} }} {f(x)dx} }}{{x_{n} - x_{n - 1} }}$$

when $f(x)$ is a monotonically increasing function, if the solution to Eq. (11) exists in the domain, then the solution is unique, the proof is shown in Online Supplementary Information. Therefore, we can quantize function $f(x)$. That is, we can quantize function $f(H)$.

### C. Iterative quantization algorithm

The variable K can be calculated using Eq. (10), and the iterative problem can be equivalent to a minimum value solution problem:

12 $${\text{min}}\{ \left| {{\text{g}}\left( x \right){ - }K} \right|\}$$

Therefore, the solution to $x$ in formula (12) is $x_{n + 1}$, and the solution to formula (12) is the solution to formula (13):

13 $${\text{min}}\{ \left| {2f(x_{n} ) - \frac{{\int_{{x_{n - 1} }}^{{x_{n} }} {f(x)dx} }}{{x_{n} - x_{n - 1} }} + \frac{{\int_{{x_{n} }}^{{x_{n + 1} }} {f(x)dx} }}{{x_{n + 1} - x_{n} }}} \right|\}$$

Intuitively, the quantization point is selected in the interval $\left[ {0,2\pi } \right]$ because the value of the H component is $\left[ {0,2\pi } \right]$. First, the initial value of the quantization interval is $x_{{{\text{n}} - 1}} = x_{0} = 0$, $x_{n} = 0$, $x_{n + 1} = 2\pi$, $n = 1$, and $a_{\min } = \max (f(H))$; second, the point $x$ that meets the conditions of formula (12) in the quantization interval $\left[ {0,2\pi } \right]$ is found, and the value $a_{{{\text{min}}}} = a$ is replaced by $x_{n + 1} = x$; third, $x = x + \Delta$; the second step is repeated until $x > = 2\pi$, then the next quantization point $x_{n + 1}$ is found; fourth, the starting value of quantization interval is updated, $x = x_{n + 1} + \Delta$, $x_{n - 1} = x_{n}$, $x_{n} = x_{{{\text{n}} + {1}}}$, $x_{n} = x_{{{\text{n}} + {1}}}$, and $a_{\min } = \max (f(H))$. The process continues to find the next quantization point $x_{n + 1}$ in the interval $\left[ {x,2\pi } \right]$. The iterative process algorithm is as follows:
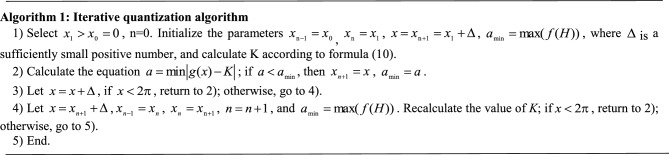


For a given value $x_{1}$, the next quantization point $x_{2}$ is calculated using the iterative algorithm, and the number of searches required by the algorithm is ${{\left( {2\pi - {\text{ x}}_{1} } \right)} \mathord{\left/ {\vphantom {{\left( {2\pi -{\text{x}}_{1} } \right)} \Delta }} \right. \kern-0pt} \Delta }$. Similarly, when quantization point $x_{n + 1}$ is found, the number of searches required by the algorithm is ${{\left( {2\pi -{\text{ x}}_{n} } \right)} \mathord{\left/ {\vphantom {{\left( {2\pi- {\text{x}}_{n} } \right)} \Delta }} \right. \kern-0pt} \Delta }$. Therefore, the number of searches required by the iterative algorithm for the quantization interval is $\sum\limits_{n = 1}^{N - 1} {(2\pi - x_{n} )/\Delta }$. When $x_{1}$ is changed, the above process must be repeated.

### D. Dichotomy quantization algorithm

The complexity of the above iterative algorithm is affected by the parameter $\Delta$. If the algorithm accuracy is high, the value of $\Delta$ should be small; otherwise, it will lead to a large amount of calculation. However, if the algorithm accuracy is low, the calculated quantization points are not good enough, and it will even cause greater errors in subsequent iterations. To solve the contradiction between the accuracy and the calculation error caused by the parameter $\Delta$, it is necessary to further study a more effective algorithm solution. So, we propose another dichotomy quantization algorithm. We create the equation:

14 $$Y(x) = g(x) - K$$

Clearly, the function $Y(x)$ is a monotonically increasing function. The derivative is taken on both sides of Eq. (14), and the equation can be expressed as $Y^{^{\prime}} (x) = g^{^{\prime}} (x)$; then.$g^{^{\prime}} (x) = \frac{{\int_{{x_{n} }}^{x} {[f(x) - f(t)]dt} }}{{(x - x_{n} )^{2} }}$. Because the function $f(x)$ is a monotonically increasing function, when $x > t$, $f(x) - f(t) > 0$, ${\text{g}}^{^{\prime}} (x) > 0$, and $Y^{^{\prime}} (x) > 0$, therefore, the function $Y(x)$ is a monotonically increasing function. Since $Y(x)$ is a monotonically increasing function, the extreme value problem of formula (14) can be transformed into a problem of solving the equation $Y(x) = 0$. Since $Y(x)$ satisfies formula (13), we can obtain:

15 $$Y(x)\left\{ {\begin{array}{*{20}c} { < 0 \, ,} \\ { = {0 },} \\ { > {0 },} \\ \end{array} } \right.\begin{array}{*{20}c} {{\text{ x < x}}_{{\text{n + 1}}} } \\ {{\text{ x}} = {\text{x}}_{{\text{n + 1}}} } \\ {{\text{ x}} > {\text{x}}_{{\text{n + 1}}} } \\ \end{array}$$

The dichotomy quantization algorithm can be used to find the quantization points $x_{n + 1}$, $n = 1,2 \cdots ,N - 1$ on the interval $[x_{1} ,2\pi ]$, and the process of dichotomy quantization is as follows:
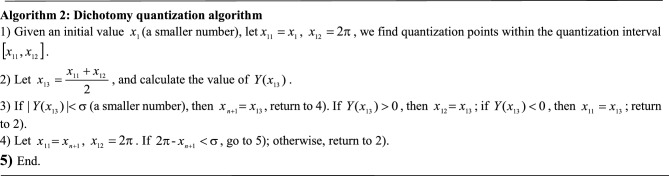


### The HSI color quantization algorithm

#### Quantize a color image in the HSI color space

According to the R, G and B components of an input RGB image, the color quantization process for the HSI color space is as follows. First, construct the $H$ component’s function $f(H)$ using the single-valued monotonic function transformation of the H component algorithm proposed of section “Methods-A”. Second, calculate the quantization point $x_{n}$ using the dichotomy quantization algorithm of dichotomy quantization algorithm of Section "Methods-D". Third, obtain the quantization value $y_{n}$ of function $f(H)$ in each quantization point, $n = 0,1,2,...,N - 1$, N represents the number of quantization points in the color space, n represents the variable the number of quantization points, using Eq. (7) with quantization point $x_{n}$. The quantization value $y_{n}$ is reshaped as follows:

16 $$Z_{n} = \left\lfloor {My_{n} /9.0 + 0.5} \right\rfloor$$

Here, $Z_{n}$ is an unsigned integer, and is the value of the $H$ component in the HSI space, $y_{n}$ is the value of a quantized point corresponding to function $f(H)$, *M* is a positive integer, ⌊ ⌋ rounds down, the number 9 is the absolute value of the value range of the function, and the number 0.5 is convenient for the subsequent experimental operation.

The calculation method for the *S* and *I* components in the HSI space is consistent with the S-SC algorithm^26^, and the S and I components are normalized before quantization. The quantization value is consistent with the quantization result of the H component; that is, the quantization table of the H component is used as the quantization table of the S and I components.

#### Generation of an RGB image

The reconstructed RGB image is obtained using formulas (17)–(19), and $Z_{n}$ needs to be inverted and shifted. This operation is equivalent to returning the N-level quantized value back to the $H^{\prime}$ transformation scale^26^*.*

When $Z_{n} \in [0,3)$, it is equivalent to $H \in \left[ {0,{{2\pi } \mathord{\left/ {\vphantom {{2\pi } 3}} \right. \kern-0pt} 3}} \right)$, and the R, G, and B components of the quantized image are as follows:

17 $$\left\{ \begin{gathered} B = I(1 - S) \hfill \\ R = I(1 + S(2 - Z_{n} )) \hfill \\ G = 3I - (B + R) \hfill \\ \end{gathered} \right.$$

(b) When $Z_{n} \in [3,6)$, it is equivalent to $H \in \left[ {{{2\pi } \mathord{\left/ {\vphantom {{2\pi } 3}} \right. \kern-0pt} 3},{{4\pi } \mathord{\left/ {\vphantom {{4\pi } 3}} \right. \kern-0pt} 3}} \right)$, and the R, G, and B components of the quantized image are as follows:

18 $$\left\{ \begin{gathered} R = I(1 - S) \hfill \\ G = I(1 + S(5 - Z{}_{n})) \hfill \\ B = 3I - (R + G) \hfill \\ \end{gathered} \right.$$

(c) When $Z_{n} \in [6,9)$, it is equivalent to $H \in \left[ {{{4\pi } \mathord{\left/ {\vphantom {{4\pi } 3}} \right. \kern-0pt} 3},2\pi } \right)$, and the R, G, and B components of the quantized image are as follows:

19 $$\left\{ \begin{gathered} G = I(1 - S) \hfill \\ B = I(1 + S(8 - Z_{n} )) \hfill \\ R = 3I - (G + B) \hfill \\ \end{gathered} \right.$$

## Results

In this section, we first provide a detailed description of the dataset and the experimental environment. Then we provide the results of the effect of different parameters. At last, we provide the results of quantization images with the proposed dichotomy quantization algorithm, which is compared with other quantization algorithms.

### Experimental environment

In our experiment,, the quantization table of H component is coded in C, and subsequent simulation is executed by MATLAB 2019 on a PC running the Windows 10 operating system with 16 GB of RAM and an AMD Ryzen 5 3500 processor (2.1 GHz).

### Quantization analysis

The initial value $x_{1}$ is necessary for the calculation of the quantization point. The parameters $\Delta$, $\sigma$, N, and $x_{1}$ greatly influence the quantization results, so we analyze the effect of these parameters.

#### Effect of the N and $x_{1}$ parameters

The quantization points are calculated by the iterative algorithm, and the number of quantization points N is affected by the iteration step size $\Delta$ and the initial value of the quantization point $x_{1}$. The simulation results show that when the iteration step is constant ($\Delta$ < $x_{1}$, where $\Delta$ is a sufficiently small number), the larger the value of $x_{1}$ is, the smaller the number of quantization points N, and the smaller the value of $x_{1}$ is, the greater the number of quantization points N. When $x_{1}$ changes within a certain range, the quantization number N does not change much, but if the value of $x_{1}$ is greater than a certain value or less than a certain value, the value range of N changes greatly. Taking a value range of $x_{1}$ from 0.01 to 0.1 as an example, the value of $\Delta$ is 0.0001, and the value of $\sigma$ is 0.0001. The relationship between $x_{1}$ and N is shown in Table 1. When the value range of $x_{1}$ is 0.03 to 0.1, $x_{1}$ is decreasing, and the value of N gradually increases and when the value range of $x_{1}$ is less than 0. 03, the value of N increases and changes greatly.

**Table 1:** The number of quantized points.

N	x_1_						
	0.1	0.05	0.04	0.03	0.02	0.15	0.01
Iterative algorithm	38	73	90	119	176	243	352
Dichotomy algorithm	37	73	88	118	176	243	367

The quantization points are calculated using the dichotomy algorithm, and the number of quantization intervals *N* is affected by the termination condition and the initial value of the quantization point $x_{1}$. To verify the performance of the dichotomy method, the relationship between $x_{1}$ and the number of quantization points *N* and the relationship between the value of the termination condition $\sigma$ and the quantization number *N* are simulated. The results show that the value of $x_{1}$ affects the size of the number of quantization points *N*. In general, the larger the value of $x_{1}$ is, the smaller the number of quantization points *N* and the smaller the value of $x_{1}$ is, the greater the number of quantization points *N*. When $x_{1}$ changes within a certain range, the quantization interval value *N* does not change much, but if $x_{1}$ is greater than a certain value or less than a certain value, the value range of *N* changes greatly. Taking a value range of $x_{1}$ from 0.01 to 0.1 as an example, the relationship between $x_{1}$ and the quantization point *N* is shown in Table 1. When the value of $x_{1}$ is between 0.03 and 0.1, the value of *N* increases and changes little and when the value is less than 0.03, the value of *N* increases and changes greatly.

#### Effect of the $\Delta$, $\sigma$, and N parameters

**Figure 3:**
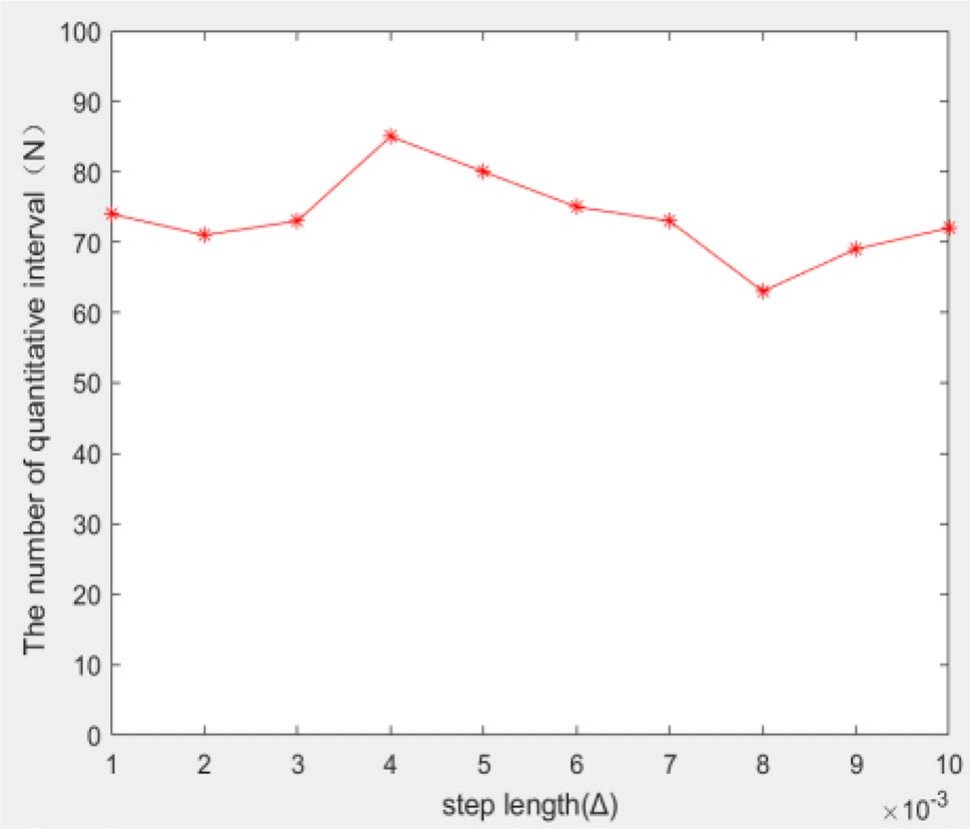
Relationship between Δ and N.

While the quantization points are calculated using the iterative algorithm, the quantization step size $\Delta$ also affects the value of the quantization points number N. When the value of $x_{1}$ is constant, and the step size $\Delta$ is less than $x_{1}$, the value of the step size $\Delta$ has little effect on the number of quantization points N. As the step size $\Delta$ changes, the number of quantization points N fluctuates in a certain range, but the range is not large. Taking $x_{1}$ = 0.05 as an example, the result is shown in Fig. 3, the step size $\Delta$ ranges from 0.01 to 0.001, and the number of quantization points N fluctuates near.

In practical applications, when N is a specific value, $x_{1}$ and $\Delta$ must be selected appropriately.

**Figure 4:**
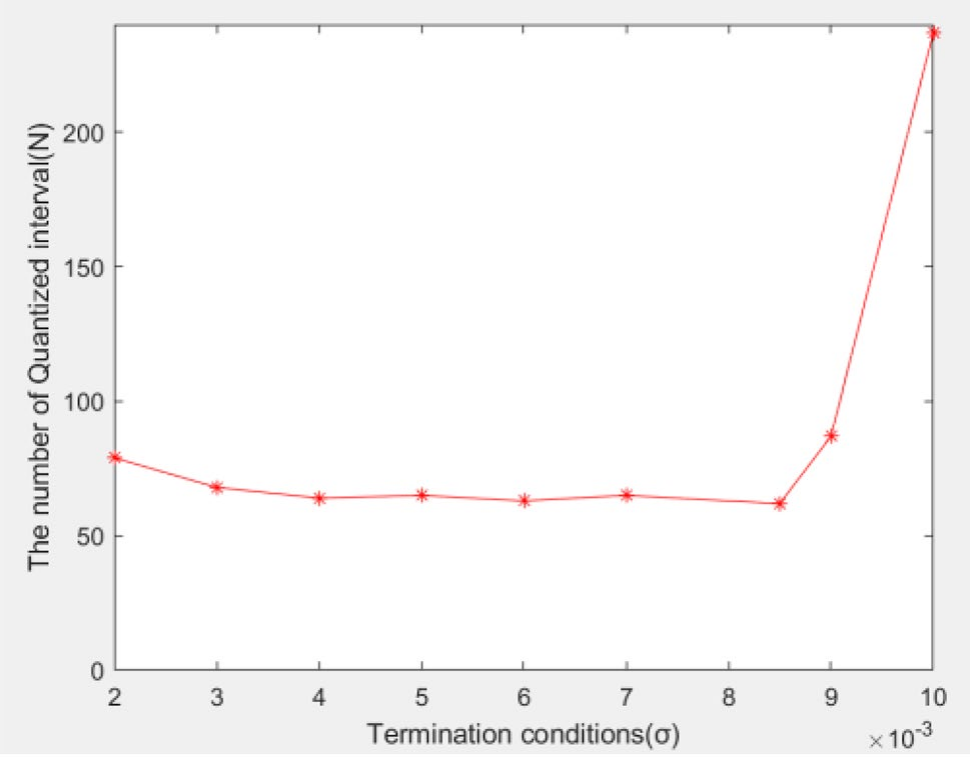
Relationship between $\sigma$ and N.

When the quantization point is calculated using the dichotomy method, the value of the termination condition $\sigma$ will also affect the value of *N* to a certain extent. When the value of $x_{1}$ is constant and the termination condition $\sigma$ changes within a certain range ($\sigma$ is small enough), the value of *N* does not change much; however, if the termination condition $\sigma$ is greater than a certain value or less than a certain value, the value range of *N* changes greatly. Taking the value of $x_{1}$ as 0.05 as an example, the result is shown in Fig. 4. The termination condition $\sigma$ for calculating the quantization point varies between 0.002 and 0.009, and the value of *N* does not change much. When $\sigma$ is set to 0.01, the number of quantization intervals *N* has a relatively large change. In practical applications, when *N* is a specific value, $x_{1}$ and $\sigma$ must be selected appropriately.

#### Effect of the $x_{1}$ parameter

Previous section specifies that the number of searches required by the iterative algorithm is $\sum\limits_{n = 1}^{N - 1} {(2\pi - x_{n} )/\Delta }$, and the number of algorithm searches is related to the iteration step size. However, the number of searches required to quantize using the dichotomy method is lower. We use the iterative and dichotomy algorithms to quantize the interval $[0,2\pi )$, and the simulation results of the number of iterations are shown in Table 2. When the value of $x_{1}$ is the same, the number of iterations of the dichotomy algorithm is much less than that of the iterative algorithm, and the precision value is 0.0001. Therefore, the dichotomy algorithm is less complex than the iterative algorithm.

**Table 2:** Number of iterations for quantization points.

x_1_	Iterative algorithm	Dichotomy algorithm
0.1	904917	485
0.05	1735387	883
0.04	2101056	1095
0.03	2866205	1480
0.02	4246815	2183
0.015	5620452	2800
0.01	8560153	4283
0.009	9898619	4806

### Quantization simulation results

**Figure 5:**
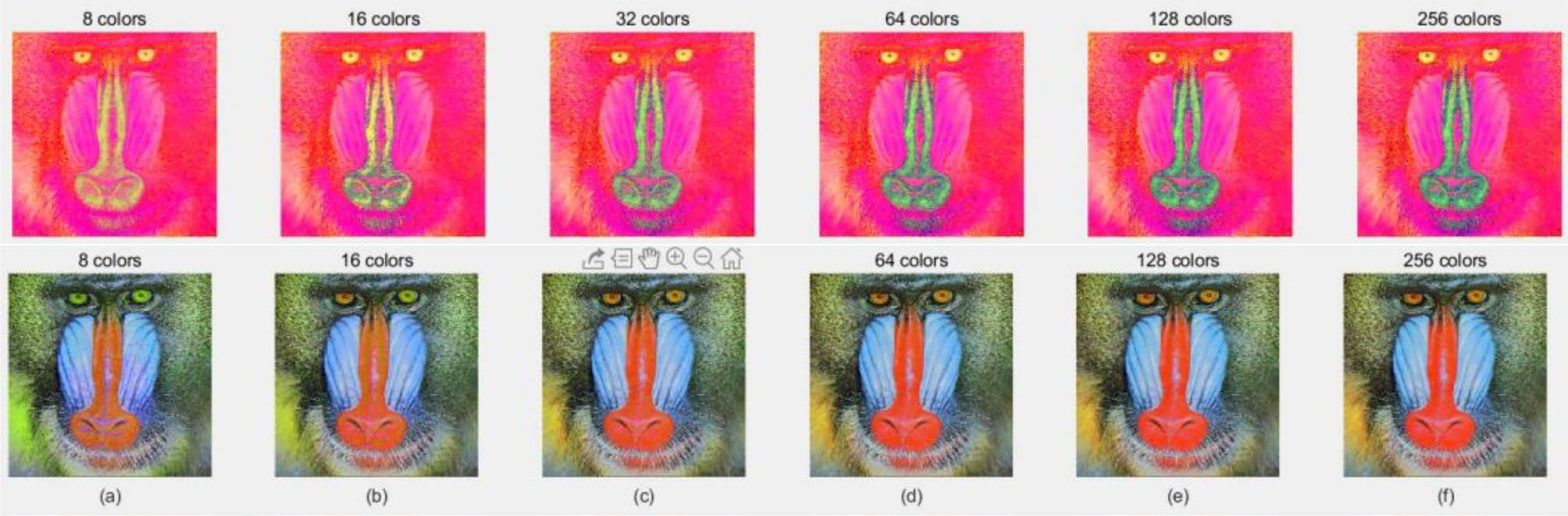
The quantized HSI image and the reconstructed image.

We use the dichotomy algorithm to quantize color image. After many experiments, the value of $x_{1}$ is set to 0.01, and $\sigma$ is set to 0.001. Taking the Mandrill picture as an example, the Mandrill image is published under the CC-BY open access license. For different quantization points, the quantized HSI image and the reconstructed RGB image are shown in Fig. 5, there are obvious differences at different quantization points. When the number of quantized colors is smaller, the distortion of the HSI image is more obvious; when the number of quantized colors is different, the quality of the reconstructed image is different. When the value of N is 128, the reconstructed RGB image is very close to the original image. Compared with the original image, the MSE value of Mandrill picture is as low as 2.75, and the results are recorded in Table 3. With the gradual increase in the value of N, the reconstructed RGB image gradually becomes similar to the original image. When the value of N reaches 128, the reconstructed RGB image is very close to the original RGB image.

**Table 3:** The MSE value of each image using different quantization levels.

	N	ATCQ^16^	PSO + KM^12^	ABC + KM^12^	SFLA-CQ^12^	Proposed
Mandrill	32	758.39	373.32	373.66	416.42	43.9
	64	393.09	236.34	235.88	268.61	11.19
	128	220.56	152.43	151.37	196.42	2.75
	256	134.34	97.67	97.14	158.07	0.69
Plane	32	109.16	67.38	71.63	81.09	13.18
	64	58.3	44.53	46.34	46.89	4.01
	128	35.49	28.56	31.63	33.41	0.94
	256	23.76	20.16	20.53	20.30	0.24
Lake	32	268.93	203.94	203.29	202.55	23.89
	64	178.38	131.64	130.84	131.51	6.29
	128	118.89	85.67	86.19	86.22	1.56
	256	76.29	56.23	56.78	56.64	0.39
Peppers	32	419.31	230.88	227.26	230.14	68.05
	64	189.87	135.39	134.03	134.47	16.3
	128	116.72	84.74	83.74	83.84	4.46
	256	72.96	55.68	54.78	54.71	1.15

### Comparison with other color quantization methods

#### Comparison with other HSI quantization algorithms

There are few HSI quantization methods, the quantization methods are based on uniform quantization. For example, in literature^28^, the H component is quantized into 7 quantization points, and the S and I components are quantized into 3 quantization points. Moreover, the purpose of these few HSI quantization algorithms is not color quantization; that is, quantized color images cannot be obtained.

Therefore, to verify the performance of each algorithm, we use a uniform quantization algorithm to compare the dichotomy algorithm using the PSNR^27^. The PSNR calculation method is as follows:

20 $$PSNR = {10} \times {\text{log}}\left( {\frac{{255^{2} }}{MSE}} \right)$$

21 $$MSE = \frac{{\left( {\sum\nolimits_{{{\text{i}} = 0}}^{m} {\sum\nolimits_{j = 0}^{n} {\left( {I\left( {i,j} \right) - K\left( {i,j} \right)} \right)^{2} } } } \right)}}{mn}$$

**Figure 6:**
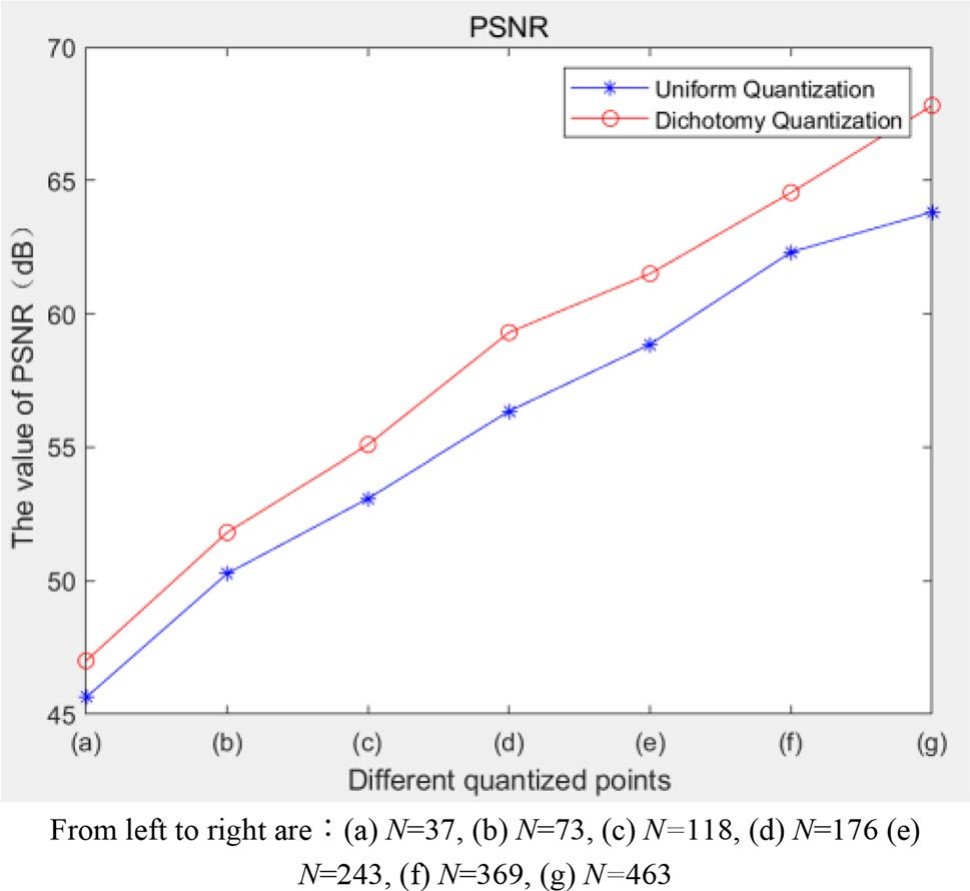
The PSNR of the uniform and dichotomy algorithms.

MSE represents the mean square error between the original image and the quantized image. I represents the original image, K represents the quantized image, m and n represent the dimension of image. The specific process of calculating the PSNR is as follows: the calculation process of the quantization colors is that the value range of R, G, and B are 0 ~ 255, and there are $256^{3}$ combinations of the values of the three components; the value of $H^{^{\prime}}$ is calculated using Eq. (1), and the value of function $f(H)$ is calculated using Eq. (3). The uniform quantization algorithm samples the H components uniformly. The PSNR is calculated for the two algorithms, taking the Lena image as an example, and the results are shown in Fig. 6. When the value of N is the same, the PSNR value of the dichotomy algorithm is higher than that of the uniform method. This is the reason why we choose the dichotomy algorithm to determine the quantization points in this paper.

#### Comparison with other RGB quantization algorithms

Since there are few image quantization algorithms in HSI color space, in order to prove the effect of our proposed quantization , we transfer the images quantized in HSI color space to RGB color space, and to compared with RGB images which are quantization by other RGB color quantization methods.

In this part, the images used for test are commonly used to test color quantification methods^29^, they are all the same dimensions of 512 × 512, the test images include Mandrill, Plane, Lake, Peppers. To compare the performance with other RGB quantization s, the number of quantization points N is set to a fixed number. Additionally, we take N as one of the conditions for the termination of the dichotomy quantization algorithm. The images are quantized to 32, 64, 128 and 256 colors, i.e., the values of N of the proposed are 32, 64, 128 and 256. The MSE of our quantization algorithms is shown in Table 3.

We choose the ATCQ^16^, PSO + KM^12^, ABC + KM^12^, SFLA-CQ^12^ to comparison, the reasons are as follows: one is that the three methods ATCQ, PSO + KM, and ABC + KM are well-known color image quantization methods and other quantization algorithms often compare with, the other is that the size of the test images of these methods is pointed out in the references^12^, compared with the number of quantization points of other quantization articles. The number of quantization points of the references^12^ is relatively rich. In addition, the SFLA-CQ in the references^12^ was published in 2019, which was relatively new in the color quantization algorithm. The MSE of the comparison RGB quantization algorithms are copied from the Tables 3 and 5 of the cited article^12^, the values of MSE are the minimum values in the different quantization points for quantizations.

The compared results are recorded in Table 3, It can be seen from the table that when the color quantization point of our proposed is 32, the MSE of Mandell image of the proposed algorithm has been as low as 43.9, however, the MSE values of ATCQ, PSO + KM, ABC + KM and SFLA-CQ are 758.39, 373.32,373.66, 416.42, of all the other four RGB quantization methods; When the quantization point is 64,128 and 256, the MSE of Mandell image has been as low as 11.19, 2.75 and 0.69 respectively. The MSE of Mandell image of the proposed HSI quantization algorithm is much lower than that of other methods based on RGB color space quantization algorithms. Similarly, the same lower MSE results are obtained in the test of the pictures Plane, Lake and Peppers than other compared quantization points in the quantization points 32, 64, 128 and 256.

It is worth emphasizing that the advantage of this proposed quantization algorithm provides a new idea for color quantization methods, which can be used by researchers in HSI color space.

## Conclusion

In this paper, we propose a new color quantization algorithm for the HSI color space. First, to construct a single-valued monotonic function of the H component, the sector information of the H component is removed. Then, based on the analysis of this function, we propose an iterative quantization algorithm, which can calculate the next quantization point from the obtained quantization points. The iterative quantization algorithm is improved using a dichotomy algorithm, which can effectively reduce the complexity of the iterative algorithm. The simulation results show that the quality of the reconstructed RGB image using the dichotomy color quantization algorithm is good for randomly selected images. The speed of the proposed quantization speed is insufficient, in the follow-up work, we will study the speed of quantifying the H component to improve the efficiency of the algorithm.

## Supplementary Information

Supplementary Information.

## Data Availability

The dataset used and/or analysed during the current study available from the corresponding author on reasonable request, also download it from the following website: https://sipi.usc.edu/database/database.php?volume=misc.

## References

[1] Pérez-Delgado ML (2021). Revisiting the iterative ant-tree for color quantization algorithm. J. Vis. Commun. Image Represent..

[2] Farshi R (2020). Color image quantization with peak-picking and color space. Multimed. Syst..

[3] Cheng SC, Yang CK (2001). A fast and novel technique for color quantization using reduction of color space dimensionality. Pattern Recognit. Lett..

[4] Xiang Z (1997). Color image quantization by minimizing the maximum intercluster distance. ACM Trans. Graph.

[5] Hsieh IS, Fan KC (2000). An adaptive clustering algorithm for color quantization. Pattern Recognit. Lett..

[6] Patané G, Russo M (2001). The enhanced LBG algorithm. Neural Netw..

[7] Celebi ME (2011). Improving the performance of k-means for color quantization. Image Vis. Comput..

[8] Omran MG, Engelbrecht AP, Salman A (2005). A color image quantization algorithm based on particle swarm optimization. Inform.

[9] Ozturk C, Hancer E, Karaboga D (2014). Color image quantization: A short review and an application with artificial bee colony algorithm. Inform.

[10] Pérez-Delgado ML (2019). The color quantization problem solved by swarm-based operations. Appl. Intell..

[11] Pérez-Delgado ML, Román Gallego JÁ (2020). A two-stage method to improve the quality of quantized images. J. Real-Time Image Proc..

[12] Pérez-Delgado ML (2019). Color image quantization using the shuffled-frog leaping algorithm. Eng. Appl. Artif. Intell..

[13] Pérez-Delgado ML (2020). Color quantization with particle swarm optimization and artificial ants. Soft Comput..

[14] Huang S (2021). An efficient palette generation method for color image quantization. Appl. Sci..

[15] Pérez-Delgado ML (2018). An iterative method to improve the results of Ant-tree algorithm applied to colour quantisation. Int. J. Bio-Inspir. Comput..

[16] Pérez-Delgado ML (2015). Colour quantization with Ant-tree. Appl. Soft Comput..

[17] Hou G, Pan Z, Huang B, Wang G (2018). Hue preserving-based approach for underwater colour image enhancement. IET Image Process..

[18] Dandawate YH, Joshi MA, Chitre AV (2008). Quality analysis of color images compressed with enhanced vector quantizer designed using HSI color space. Proc. Int. Conf. Comput. Intell. Multimed. Appl. ICCIMA.

[19] Chen S, Feng R, Zhang Y (2019). Aerial image matching method based on HSI hash learning. Pattern Recogn. Lett..

[20] Guo Y, Zhang Z, Yuan H, Shao S (2019). Single remote-sensing image dehazing in HSI color space. J. Korean Phys. Soc..

[21] Ma J, Fan X, Yang SX, Zhang X (2018). Contrast limited adaptive histogram equalization-based fusion in YIQ and HSI color spaces for underwater image enhancement. Int. J. Pattern Recogn..

[22] Zhang W, Liang J, Ren L (2017). Fast polarimetric dehazing method for visibility enhancement in HSI colour space. J. Opt..

[23] Ma S, Ma H, Xu Y, Li S (2018). A low-light sensor image enhancement algorithm based on HSI color model. Sensors-Basel.

[24] Siddiqui F (2019). FPGA-based processor acceleration for image processing applications. J. Imaging.

[25] Shuai Y, Ying T, Aries AB (2011). Clothing matching for visually impaired persons. Technol. Disabil..

[26] Zhi S, Cui Y, Deng J (2020). An FPGA-based simple RGB-HSI space conversion algorithm for hardware image processing. IEEE Access.

[27] Huang H, Huang S (2020). Fast hole filling for view synthesis in free viewpoint video. Electronics.

[28] Zhou LB, Huang S (2013). Image dimension reduction based on HSI color model. Mod. Electron. Tech..

[29] Weber, A. USC-SIPI image database. https://sipi.usc.edu/database/database.php?volume=misc.

