# Supplementary material for: A dichotomy color quantization algorithm for the HSI color space
Source: Sci Rep. 2023 May 19;13:8135. doi: 10.1038/s41598-023-34977-0 (PMC10199057; doi:10.1038/s41598-023-34977-0)
Supplement: Supplementary file 1 — Supplementary Information. [file 41598_2023_34977_MOESM1_ESM.docx]

**Appendix I**

**Theorem 1**: The function$f(H)$ is a monotonically increasing and continuous function in the interval $[0,2\pi)$.

Proof: Let

$$f_{1}(H)=2-\frac{\cos H}{\text{cos(}\pi/3-H\text{)}},H\in[0\text{，}2\pi/3)$$

$$f_{2}(H)=5-\frac{\text{cos(}H-2\pi/3\text{)}}{\text{cos(}\pi-H\text{)}},H\in[2\pi/3\text{，}4\pi/3)$$

$$f_{3}(H)=8-\frac{\text{cos(}H-4\pi/3\text{)}}{\text{cos(}5\pi/3-H\text{)}},H\in[4\pi/3\text{，}2\pi)$$

When $H=2\pi/3$, the following can be derived:$f_{1}\left((2\pi/3)^{-}\right)=f_{2}\left((2\pi/3)^{+}\right)$. When $H=4\pi/3$, the following can be derived:$f_{2}\left((4\pi/3)^{-}\right)=f_{3}\left((4\pi/3)^{+}\right)$. According to the continuous correlation definition of the function, the function $f(H)$ is continuous in the interval $[0,2\pi)$.

Next, the monotonically increasing derivative of function$f_{1}(H)$ is proven, and the derivative of function$f_{1}(H)$ is as follows:

$$\begin{matrix} f'_{1}(H)=\frac{\sin H\cos(\pi/3-H)-\text{(}-\cos H\text{)}\sin(\pi/3-H)}{\cos^{2}(\pi/3-H)} \\ =\frac{\sin(H+\pi/3-H)}{\cos^{2}(\pi/3-H)}=\frac{\sin(\pi/3)}{\cos^{2}(\pi/3-H)} \end{matrix}$$

again$∵H\in[0,2\pi/3)$,

$$\therefore0.5\leq\cos(\pi/3-H)\leq1$$

$\Rightarrow f_{1}^{'}(H)=\frac{\sin(\pi/3)}{\cos^{2}(\pi/3-H)}>0$ (1)

Therefore, $f_{1}(H)$ is monotonically increasing in the interval$[0\text{，}2\pi/3)$, which means it is monotonously increasing in the interval $[0\text{，}2\pi/3)$ of $f(H)$.

Similarly, the following can be obtained:

$f_{2}^{'}(H)=\frac{\sin(\pi/3)}{\cos^{2}(\pi-H)}>0$ (2)

$f_{3}^{'}(H)=\frac{\sin(\pi/3)}{\cos^{2}(5\pi/3-H)}>0$ (3)

It can be concluded that the function $f(H)$ is also monotonically increasing on the interval $[2\pi/3,4\pi/3)$and interval $[4\pi/3,2\pi)$.

In summary, the function $f(H)$ is derivable in the interval $[0,2\pi)$, and when $H\in[0,2\pi)$, there is always $f'(H)>0$. It can be concluded that the function$f(H)$increases monotonously in the interval $[0,2\pi)$, which is a monotonically increasing function. Therefore, it is a monotonically increasing function of $f(H)$, and **Theorem 1** is proved.

**Theorem 2:** When $f(x)$ is a monotonically increasing function, if the solution to formula (11) (in article) exists in the domain, the solution is unique.

Proof: Formula (16) (in the article) is deformed, and we can obtain:

$K=2f(x_{n})\text{-}\frac{\int_{x_{n-1}}^{x_{n}} f(x)dx}{x_{n}-x_{n-1}}=f(x_{n})+\frac{\int_{x_{n-1}}^{x_{n}} [f(x_{n})-f(x)]dx}{x_{n}-x_{n-1}}$ (4)

Since it is a monotonically increasing function of $f(x)$, when $f(x_{n})>0$, $K>f(x_{n})>0$. The derivative of $g(x)$ is taken, and we can obtain:

$g'(x)=\frac{\int_{x_{n}}^{x} [f(x)-f(t)]dt}{\left(x-x_{n}\right)^{2}}$ (5)

Function $f(x)$ is a monotonically increasing function; when $x>t$, $f(x)-f(t)>0$, which is equivalent to $g'(x)>0$. It has been proven that K is greater than 0, so equation $g(x)=K$ has one solution at most, and the formula (4) can solve at most one solution. When quantization interval $x_{n}$, $x_{n+1}$ are known and $f(x)$ is a monotonically increasing function, then not only the solution to the equation $g(x_{n+1})=2f(x_{n})-\frac{\int_{x_{n-1}}^{x_{n}} f(x)dx}{x_{n}-x_{n-1}}$ $=\frac{\int_{x_{n}}^{x_{n+1}} f(x)dx}{x_{n+1}-x_{n}}$ exists but also it is the only one, **Theorem 2** is proved.
